# A Tunable Silk Hydrogel Device for Studying Limb Regeneration in Adult *Xenopus Laevis*

**DOI:** 10.1371/journal.pone.0155618

**Published:** 2016-06-03

**Authors:** Anne Golding, Justin A. Guay, Celia Herrera-Rincon, Michael Levin, David L. Kaplan

**Affiliations:** 1 Department of Chemical and Biological Engineering, Tufts University, Medford, Massachusetts, United States of America; 2 Department of Biology, Center for Regenerative and Developmental Biology, Tufts University, Medford, Massachusetts, United States of America; 3 Department of Biomedical Engineering, Tufts University, Medford, Massachusetts, United States of America; Université de Technologie de Compiègne, FRANCE

## Abstract

In certain amphibian models limb regeneration can be promoted or inhibited by the local wound bed environment. This research introduces a device that can be utilized as an experimental tool to characterize the conditions that promotes limb regeneration in the adult frog (*Xenopus laevis*) model. In particular, this device was designed to manipulate the local wound environment via a hydrogel insert. Initial characterization of the hydrogel insert revealed that this interaction had a significant influence on mechanical forces to the animal, due to the contraction of the hydrogel. The material and mechanical properties of the hydrogel insert were a factor in the device design in relation to the comfort of the animal and the ability to effectively manipulate the amputation site. The tunable features of the hydrogel were important in determining the pro-regenerative effects in limb regeneration, which was measured by cartilage spike formation and quantified by micro-computed tomography. The hydrogel insert was a factor in the observed morphological outcomes following amputation. Future work will focus on characterizing and optimizing the device’s observed capability to manipulate biological pathways that are essential for limb regeneration. However, the present work provides a framework for the role of a hydrogel in the device and a path forward for more systematic studies.

## Introduction

Regeneration is the process of restoration, renewal, and growth that an organism undergoes to survive in response to an injury [1]. All living things regenerate to some degree, as seen in wound healing, embryonic development, and even adaptive biology [1]. Even higher organisms such as mammals are capable of some complex regeneration. Adult mice are able to regenerate the tip of the most distal phalange bone of their finger [2]. Ideally, the resultant tissue would have the same functional and structural capability as in the pre-injured state. However, for adult mammals, this level of regeneration is the exception not the rule. Even healed skin that looks flawless at the surface only regains about 80% of the pre-injured mechanical strength [3]. Mended bones are prone to fracture due to a loss of structural integrity from the healing process [4]. When the tip of a mouse finger is amputated a new one can grow back, however, the resultant fingertip contains bone that is less structured than the original [2].

In contrast to the outcomes during regeneration, during development, humans and other mammals are capable of complete regeneration [5]. Generally, both types of regeneration follow the same healing cycle and utilize the same biological pathways[5]. The differences tend to be in the cell types and local cellular environment that partake in this process [1,6–10]. This is in part due to the fact that as mammals go through development they gain different biological functions that are necessary for survival at the price of losing this complete regenerative capability [11]. One of the major goals in the field of bioengineering is to utilize biomaterials to help provide the needed environmental cues to overcome these road blocks that prevents complete regeneration. This type of strategy has been applied in both *in vitro* and *in vivo* models [12]. While there has been headway using this method, there is still a large discrepancy between the resultant regenerative tissue obtained here and the outcomes observed in embryonic tissue regeneration [5].

While complete tissue or organ regeneration does not naturally occur in mammals, it can occur in amphibians, fish, and other lower organisms [13]. Two of the most commonly used animal models in the study of regeneration are the tail fin regeneration in zebrafish and limb regeneration in *Ambystoma mexicanum* axolotl [1]. But, the regenerative mechanisms used in these simpler animal models are different than what has been observed in the mouse fingertip model [2]. Axolotl and zebrafish use biological pathways that are more akin to what is observed in prenatal development, while mammalian models utilize a more adaptive wound healing response [2,14]. So, while the information obtained using these simpler models provides insight to what biological pathways are present in complex regeneration, it can very difficult or nearly impossible to directly apply this information to what is observed in mammals.

The frog, *X*. *laevis*, is similar to both the axolotl and zebrafish in that it can completely regenerate limbs after amputation. However, an important distinction that *sets X*. *laevis* apart from the other two animal models is that this innate regenerative ability wanes as the animal develops. After about stage 61 of development, tissue growth does not provide the morphological characteristics of a limb, rather the animal forms a cartilage spike [15]. Another advantage of this animal model compared to the traditional urodele model is that the cells used in the tissue reconstruction process are not progenitor cells [2]. Rather, the tissue is composed of fibroblast cells. This makes the frog model more applicable to the mouse regenerative model, which also utilizes fibroblasts in the same manner for fingertip regeneration [2]. These combined aspects make the frog model a needed bridge between the simpler but less applicable axolotl and zebrafish models and the more relevant but complex mouse fingertip regenerative model [16].

Typically, two experimental setups are utilized for regenerative animal models. The first is a gain/loss of function model. This type of model will either inhibit or activate a given receptor that is known to promote or inhibit the biological pathway being investigated. By “rescuing” the animal from the initial gain/loss a direct correlation can be attained of that given pathway to the observed change in biological outcomes [17]. However, these models tend to be taken out of context of the overall regenerative event and therefore are not always applicable [17]. The other type of experimental setup takes the opposite approach and uses a cocktail to promote regeneration. This cocktail includes a variety of known pro-regenerative stimuli (e.g. growth factors, drugs, progenitor cells, genetically engineered or surgically modified animals [18]) which will almost certainly result in some degree of change in biological outcome [18]. In theory after the desired result is achieved then different factors in the cocktail would be removed individually to assess the role of that particular component in the final outcome. This method will provide a desired result, but not an accurate means to correlate the result to a cause, due to the heterogeneous nature the cocktail [18]. The first method emphasizes correlation of a cause and effect with little applicability while the second achieves a result but lacks the ability to correlate the result to a specific cause. A middle ground needs to be established in order to understand the mechanisms that drive regeneration in an applicable manner. Moreover, regeneration is a dynamic process which requires temporal control over inputs, which neither model can provide.

Devices have also been fabricated to help with understanding regenerative processes [19,20]. Previous reported work from this research group presented a similar device design to promote fingertip regeneration in mice [20]. This “proof-of-concept” device was attached to the stump of an amputated finger and delivered a cocktail of different regenerative compounds via a solution, as well as an applied electric current, to the wound site [20]. Morphological changes were observed in the tissue near the site of amputation when the animals were administered a cocktail that contained urinary bladder matrix pepsin digests [20]. However, the device had technical limitations, such as fit, ability to achieve attachment throughout the entire experimental population, and subsequent tissue necrosis. These types of technical issues not only effect the livelihood of the animal, but also the quality of any resultant regeneration. For this device to be an effective tool for regenerative research it would need to be redesigned to overcome these technical issues. The updated device would need to be evaluated for its ability to be used effectively with the animal, as well as its capability to manipulate the local wound bed to promote regeneration. Mouse fingertip regeneration takes a long time to complete and is costly in both in time and resources to assess. Frog leg regeneration is easier to assess and takes less time. This, in conjunction with the aforementioned biological benefits makes this an excellent alternative for technical evaluation of device design.

Tissue scaffolds are one of the fundamental tools utilized in regenerative medicine. By tuning the material properties of these scaffolds, engineers can provide to a wound site the needed stimulus to promote tissue regeneration [12]. This tunability is essential for any device that would be used to study limb regeneration. Silk as a protein biomaterial is mechanically robust, has low immunogenicity, and can be used to fabricate a wide variety of materials (e.g. sponges, hydrogels, and films) that have tunable mechanical properties [21]. Furthermore, silk biomaterials can also be used for compound delivery [22–24]. In particular, a silk hydrogel would provide a hydrated environment to the wound site, which has been shown to be essential for wound healing and limb regeneration [25]. A hydrogel based reservoir would remove the need for a water-tight seal, which was a requirement of the past device, and a major contributor to necrosis [20]. Thus, this material is an excellent option for this type of device.

The goal of the presented work was to implement this new design for the *X*. *laevis* model. The proposed device has an improved mode of delivery of various factors to the amputation site. This new experimental device consisted of a holder and hydrogel and a corresponding set of analytical methods to characterize and manipulate different environmental factors required to stimulate biological pathways toward limb regeneration. This device provides a systematic approach that can streamline overall experimental processes to correlate environmental inputs to gross anatomical changes as a quantitative starting point to explore the molecular mechanisms that drive regeneration.

## Methods and Materials

### Device Fabrication

**Fig 1:**
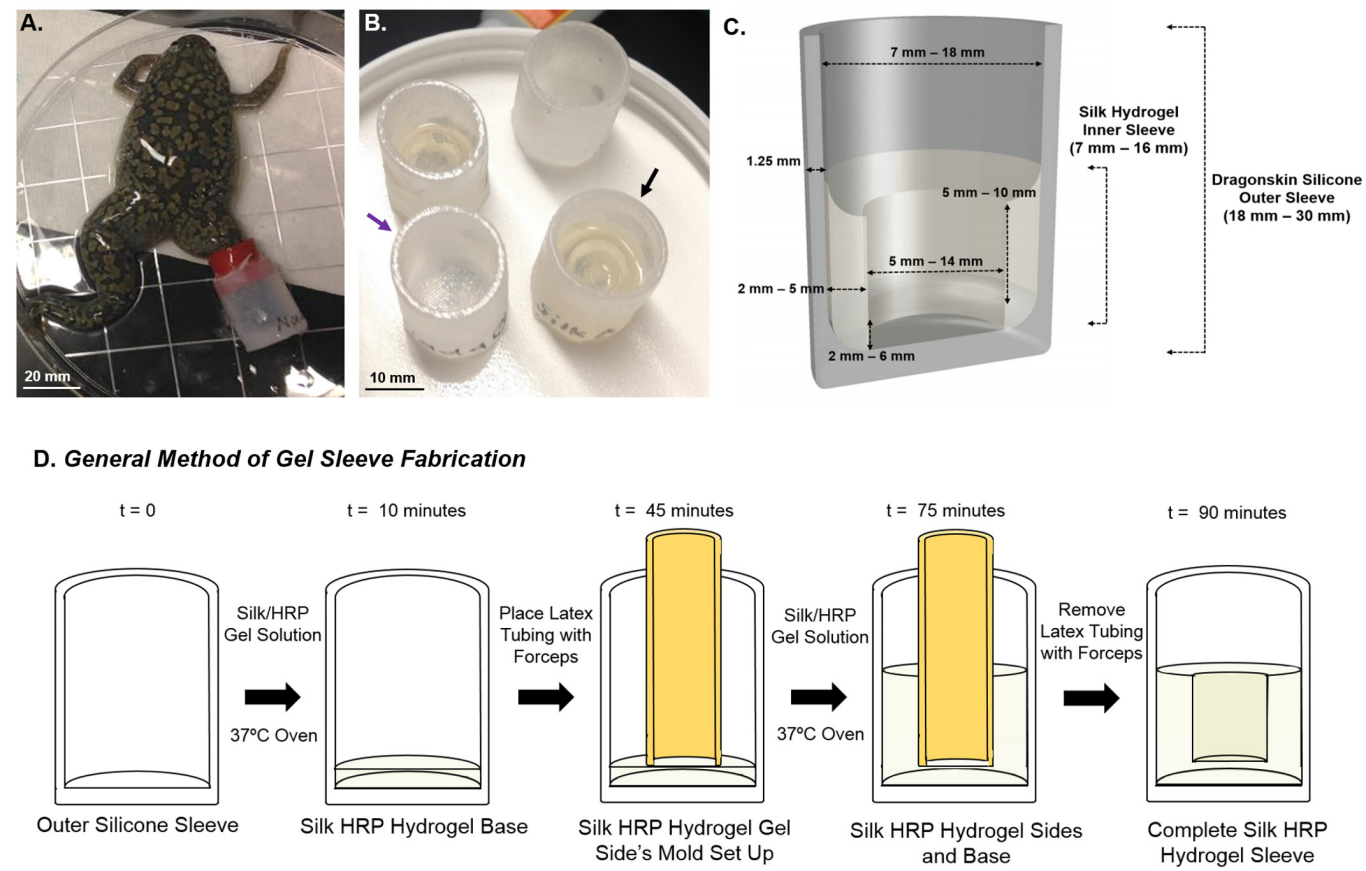
Design and Fabrication Process for the Device. (A) Image of the device attached to the animal using a silicone wrap for attachment. (B) Final version of the soft Dragonskin. The purple arrow points out a device with an outer sleeve with no inner hydrogel insert, while the black arrow points out an experimental device with a hydrogel. (C) Schematic of the outer silicone and silk hydrogel insert. (D) Diagram of the hydrogel insert fabrication process.

The device (Fig 1) was composed of three separate components: 1) an outer sleeve made from Dragonskin Very Fast 10A silicone rubber (Smooth On, Easton, PA), 2) a rubber strip (McMasterCarr, Robbinsville, NJ) used to attach the device to the amputated limb, and 3) a hydrogel insert used to deliver the different types of biochemical (or mechanical) stimuli applied to the wound bed. The size of the device can be modified to fit each animal, but generally sizes which range from an inner diameter of 7 to 18 mm were pre-prepared. The thickness of the walls of the device ranged from 1.25 to 1.5 mm, and the overall length of the outer sleeve was 18 to 30 mm long, but can be trimmed to fit the animal upon attachment. The volume of the hydrogel depends upon the size of the device but can range from .3 to 1.5 mL. The outer sleeve of the device was composed of a soft two-part (part A and part B) silicone polymer Dragonskin. Polymerization was achieved by mixing the two components in a 1:1 mass ratio. This was then poured into a 3D printed acrylonitrile butadiene styrene mold (Dimension Elite, Stratasys, Edina MN) and allowed to solidify for an hour. Each ABS mold was coated with an acrylic resin (Rust-Oleum, Vernon Hills, IL) to prevent contamination of the outer sleeve of the device by possible leechables from the ABS mold. Due to the nature of the silicone elastomer any contamination from the mold would result in inhibition of the curing process. Thus, a successfully cured outer sleeve would indicate that there was minimal or no contamination from the ABS mold.

Older generations of the device had different outer sleeves and followed different fabrication methods. The first generation device had a fabric outer sleeve (S1 Fig) composed of Ace bandage (Fischer Scientific, Pittsburgh, PA) that was stitched into the shape of the sleeve. At the base of the sleeve a hole was cut out and the ends sealed with paraffin wax (Fischer Scientific, Pittsburgh, PA) to prevent the cloth from unraveling. This inserted hole was then sandwiched between a piece of clear silicon and a rubber O-ring (McMasterCarr, Robbinsville, NJ). The three layers were then stitched together and sealed with an acrylic epoxy (McMasterCarr, Robbinsville, NJ). Using the rubber O-ring as a mounting platform, a short piece of tygon tubing (McMasterCarr, Robbinsville, NJ) was attached to the sleeve using a two-part medical grade epoxy, EPO-TEK 301 (Epoxy Technologies Billerica, MA). A 3% (w/v) silk enzymatically crosslinked horseradish peroxidase (HRP) hydrogel base was then hydrogelled in the tygon tubing. The next generation consisted of a thick rubber base, a silicone tube cuff, and a larger tygon tube reservoir (McMasterCarr, Robbinsville, NJ). These components were glued together using the two-part medical grade epoxy mentioned previously (S1 Fig).

#### Silk Processing

The method used in silk material processing for hydrogel formation for the device has been described previously [21]. Ten grams of cocoons from *Bombyx mori* silkworm were extracted in 4 liters of a 0.02 M sodium carbonate (Sigma-Aldrich, St. Louis, MO) solution for 45 minutes to remove the sericin. The resultant fibroin fibers were then dissolved in 9.3 M lithium bromide (Sigma Aldrich, St. Louis, MO). The final concentration of dissolved silk to lithium bromide was 1:4 volume ratio [21]. This was then placed into a 60°C oven for up to 3 hours to completely dissolve the silk. The dissolved silk and lithium bromide solution was dialyzed for three days in 2 L of distilled water (diH_2_O) with intermittent water changes. To ensure that almost all the lithium was removed from the silk solution, the dialysis water was sampled and its conductivity checked until the levels were under 2.5 μS. The silk solution was filtered to remove contaminants via ultra-centrifugation and then passed through a 0.22 μm syringe filter to sterilize. The concentration of the silk was measured by weight per volume (w/v) from the change in mass before and after complete evaporation of the water component of the solution in a 60°C oven.

#### Silk HRP Enzymatically Crosslinked Hydrogel Insert Fabrication

The silk solution was diluted to 3% (w/v), 3.5% (w/v), and 4.0% (w/v) in diH_2_O or diH2O with the addition of an, aquarium saline additive, Instant Ocean (Instant Ocean, Blacksburg, VA). The modified aquarium water will be referred to as frog water (FW). This was then enzymatically cross-linked at the tyrosine side chains using horseradish peroxidase enzyme (HRP) at 20 U/ml (Sigma-Aldrich, St. Louis, MO) with hydrogen peroxide at 0.01% overall volume (or 10 μl/1mL solution)[26]. As presented in Fig 1D, the bottom of the hydrogel insert was formed by pipetting .2 to 1 mL of the HRP, hydrogen peroxide, and silk solution into the device and then placed into a 37°C oven to accelerate the crosslinking process. The remainder of the hydrogel inserts s were then formed by placing a piece of latex tubing perpendicular to the bottom hydrogel layer of the device, and adding .1 to 1 mL of silk HRP solution into the device around the latex tubing. This system was then placed back in the 37°C oven for gelation. After the solution solidified, the tubing was removed carefully with a pair of forceps to form the hydrogel inserts. The thickness of the hydrogel insert was determined by the choice of the outer diameter of the latex cast tubing and the inner diameter of the outer silicone sleeve. The height of the base and sleeves were selected by adjusting the volume of the pre-hydrogelled solution. The equations used are below:

$$V_{b}\left[ml\right]=\left(\pi r_{b}^{2}h_{b}\right)\left[cm\right]$$ (1)

$$V_{s}\left[ml\right]=(\pi \left(r_{b}-r_{s}{)}^{2}h_{s}\right)\left[cm\right]$$ (2)

Where V_b_ was the volume of silk solution needed for the base of the sleeve, V_s_ was the volume of silk solution needed for the hydrogel inserts, r_b_ was the radius of the inner diameter of the Silicone Dragonskin sleeve, r_s_ was the radius of the outer diameter of the latex tubing used in the sleeve fabrication step, h_b_ was the desired height of the base, and h_s_ was the desired height of the sleeves. A lithium release study was performed to assess the possibility of any residual lithium in the HRP silk hydrogels from the silk processing step. 3% (w/v) silk HRP hydrogel disks were soaked in 10 mL of PBS for 24 hours with titer sampling at .5, 12, and 24 hours. Lithium was characterized using inductively coupled plasma atomic emission spectroscopy (ICP-AES) (Prodigy Series 1000, Teledyne Leeman Labs, Hudson, New Hampshire). A more detailed procedure can be found in the supplementary material (S1 File).

### Mechanical Testing of Hydrogel Materials

3% (w/v) HRP silk hydrogels were cast in 35 mm well dishes. An 8 mm biopsy punch was used to generate approximately 2.5 to 3 mm thick hydrogel disks. Mechanical compression was determined at a 30% strain at a rate of 1 mm/min using a TA Instruments RSA3 Dynamic Mechanical Analyzer (TA Instruments, New Castle, DE). For each trial, six samples were loaded onto the device, and the strain and applied force was measured. Five conditioning cycles were performed and then the sixth cycle was taken and used for analysis. The tangential modulus was determined at 5% strain and the stiffness was calculated. The sample stiffness values were averaged and then compared among multiple trials to ensure accuracy. In order to characterize the changes in the physical properties of the hydrogel insert during the time of attachment, the weight of each device and the device with the hydrogel insert were measured before and after *in vivo* studies. The device, the hydrogel, and the device together with the hydrogel were weighed pre and post attachment. A control set of devices was also placed into the housing tank at the time of attachment and retrieved at device removal. These materials were also evaluated for changes of mass and volume before and after device attachment and removal. A subset of experimental and control hydrogel inserts was allowed to soak in the treated animal water for 24 hours, which resulted in the hydrogels reaching the maximum absorption (swell, mass/volume) [27]. The swollen hydrogels were then weighed and placed in a 60°C oven for 24 hours. These dried hydrogel inserts were then weighed. All the hydrogels, unless otherwise stated, were prepared from a concentration of 3% (w/v) silk. The loss of mass was calculated by using the difference of the dried (m_d_) over the pre-attached mass (m_o_) which was than compared to the initial cast silk concentration (i.e. 3% (w/v) silk).

### Animal Care, Surgery, Device Attachment, and Removal

Animal trials followed pre-approved protocols from the Tufts University, Tufts Medical Center, and Center of Human Nutrition Research Center on Aging Institutional Animal Care and use Committee (IACUC) (2015-M2014-79 and 2015-M2014-79-Admendent A-7). The *X*. *laevis* population (Juvenile *Xenopus laevis Frogs* LM00453MX, Nasco, Fort Atkinson, WI) used in the animal trials were a mix of male and female with a total population of 64 animals. Animals were kept in 10 L tanks, fed with Purina Aquamax frog pellets (Nasco, Fort Atkinson, WI) with 12-hour light and dark cycles. All animal surgeries were performed under benzocaine induced anesthesia and all effort was made to minimize any suffering the animal might of experienced. The animals were administered 75 mg/kg of the analgesic buprenorphine (Sigma Aldrich, St. Louis, MO) after amputation and thereafter if they showed signs of pain (e.g. lethargic behavior). In extreme situations (e.g. excessive tissue damage) they were euthanized to prevent prolonged suffering. Frogs used were between 5 and 10 cm in length, and maintained in buffered Instant Ocean media at a conductivity of approximately 1650 μS and pH 7.3 that was changed daily. Animals post stage 61, length 1.5 to 2 cm snount to vent (length of the body excluding limbs) were anesthetized by soaking them a 0.5% benzocaine buffered solution for 2 to 5 minutes. The hind-limb was then amputated using a sterile #10 scalpel blade, and then the animal was allowed to rest for approximately three hours before device attachment.

The mode of attachment of the device involved securing it with a wrap (McMasterCarr, Robbinsville, NJ) about 3 to 6 cm in length followed by stitching the device directly to the animal and the wrap. To be more specific, the device was attached by first applying a rubber wrap around the amputated limb. The device was then positioned on the limb and secured to it and the wrap by four stitches (two to the limb and two to the wrap). The two suture points were placed in the lateral and medial end side of the leg stump, respectively, by sub-dermal stitches. First, the needle was inserted by the bottom lateral side of the device, from outside. The needle was then passed to the leg edge, on the opposing side equidistant to the insertion. The needle was then reversed in the needle holder and the skin and sub-dermal deep tissue was penetrated at 3 cm from the bottom of the amputation plane, just the same length than device (long between silk edge and top of the device). The same steps were repeated for the medial side of the device. After this preparation, the two sutures were pass through the bottom side of the side, from inside, introducing the stump leg in the device. The closure point was just 2–3 mm to the first existing point, in order to maximally decrease the possible tissue strangulation. Finally, a cyanoacrylate adhesive (McMasterCarr, Robbinsville, NJ), was used to further strengthen the attachment of the device to the wrap. In the cloth model, the device was secured using a zip tie (McMasterCarr, Robbinsville, NJ), while the stiff outer sleeve and the earlier soft dragonskin outer sleeve generations used just the wrap.

The device remained on the animals up to 24 hours with minimal signs of necrosis. During this time, the animal was monitored for signs of physical distress. The silicone and hydrogel inserts were removed using a pair of forceps and surgical scissors. After device removal, another device was attached to the limb or the animal was returned to the tank without a device attached. The frogs were kept alive to observe spike formation from 2 to 4 months (all reported results are normalized to the experimental group’s control animals). The frogs were euthanized by soaking them in a buffered solution with a high concentration of benzocaine (500 mg/L).

### Live Measurements and Micro-Computed Tomography (Micro-CT)

Images of the animals were taken at the point of device attachment, removal, 24 hours thereafter, and every one to two weeks after device removal. When spike formation became prominent, measurements were then taken every one to two weeks using a pair of calipers. Micro-CT images (SkyScan 1176, Bruke, Allentown, PA) were taken post mortem to observe calcified tissue formation. The scans were set to 9 μm pixel sections with a 0.5 mm AL filter. Fixed sections of leg were placed into 50 mL conical test tubes and loaded into the machine for imaging. The region of interest, size slice, and resolution of image were input via the operating software. The x-ray images were reconstructed into 3D models using SkyScan’s reconstruction software, *CTVol*. Bone density, surface area, and bone mean density were extracted using the machine’s analytical software, *CTAn*.

### Histology and Immunohistology

After micro-CT imaging, fixed limbs/spikes were decalcified in Richard-Allan Scientific Decalcifying Solution (Fischer Scientific, Pittsburgh, PA) for two weeks at room temperature, embedded in paraffin wax (Fischer Scientific, Pittsburgh, PA), and sequentially sectioned along the longitudinal axis at 10 μm. Parallel sections were stained for both general morphological structure, by means of Hematoxylin & Eosin and Masson’s Trichrome staining, and specific immunoexpression. Spatial detection of nerve tissue, chondrogenesis, smooth muscle cells and BMP-Smad signaling were performed by immunofluorescence for N-acetylated alpha tubulin (α-tubulin) [28], collagen type II (col2) [17], alpha smooth muscle actin (SMA) [29], and phospho-Smad1/5/9 (pSMAD 1/5/9) [17], respectively. Briefly, after re-hydration, sections were permeabilized in phosphate buffer solution 0.1% Triton X-100 (PBST), enzymatic antigen was retrieved using proteinase K solution (10 μg/mL; Invitrogen 25530–049, Carlsbad, CA), and blocked with 10% goat serum in PBST for 1 hour at room temperature. Samples were then rocked overnight at 4°C with primary antibody diluted in PBST+10% goat serum. The primary antibodies were anti-acetylated alpha tubulin (1:500; Sigma-Aldrich T7451, St. Louis, MO), anti-collagen Type II (1:100; EMD Millipore Cat# MAB8887, RRID:AB_2260779, Billerica, MA), anti-SMA (1:100; Sigma-Aldrich Cat# A2547, RRID:AB_476701, St. Louis, MO) and anti-pSMAD1/5/9 (1:100; Cell Signaling Technology Cat# 13820, RRID:AB_2493181, Danvers, MA). Following this primary exposure, samples were washed three times in PBST and incubated for 2 hours at room with AlexaFluor-555 conjugated secondary antibody (1:500; Invitrogen CAT# A21422, RRID: AB_21422, Carlsbad, CA) diluted in PBST+10% goat serum. Sections were then washed six times for 15 min in PBST and photographed using a BZ-X700 series microscope (Keyence, Itasca, IL).

### Statistical Analysis

ANOVA statistical analysis was performed on the data to determine statistical significance of the different sub populations. This process was accomplished using the data analysis package added to excel (Microsoft, Seattle, WA). All trials unless otherwise indicated had at least a population of 3. Statistical significance was rated at p< 0.05, 0.01, and 0.001 and this is indicated in the figures by one, two, and three stars, respectively.

## Results

### Device Technology

The device could be attached and removed by the earlier described method for both 24 and 48 hours with minimal necrosis or constriction of the animal limbs. Various methods of device attachment were explored to provide a secure fit, and the best mode was the combination of stitching to the animal in conjunction with a wrap. This combination for attachment provided a secure fit while minimizing compression-induced necrosis. Both prolonged attachment and re-attachment were evaluated for longer term use. The 24 hour time point was established as the minimal time that the device needed to be attached to be considered a success, since this time periods allowed for adequate time to apply a range of regenerative compounds. Thus all subsequent experiments were conducted at this time point. Trials used to assess the capabilities of the device involved 3 to 18 animals per trial, with at least one trial having three consecutive days of device attachment and re-attachment (in total 36 devices). Since lithium bromide is the chaotropic used to dissolve the silk and is also a known Wnt-pathway activator, which plays a role in most regenerative processes [30], it was important to assess residuals in the hydrogels (S1 Table). All control hydrogel samples were similar to the blank. The minimal concentration for biological function is 0.5 mM or 20 ppm, thus sample contamination was not an issue in the biological results.

### Materials Characterization

**Fig 2:**
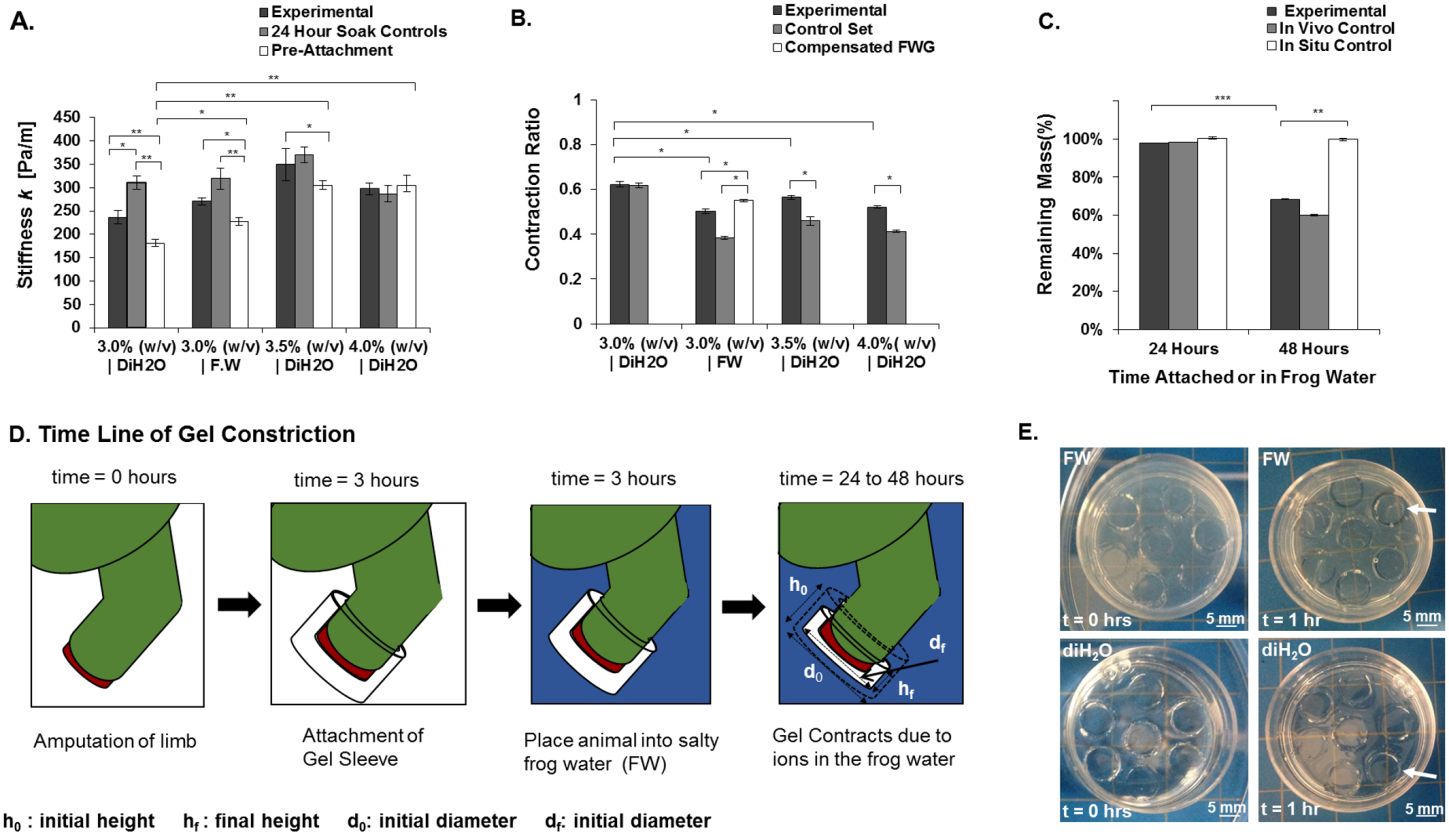
Mechanical and Material Properties of the Device. (A) Change in Stiffness of Hydrogel insert Pre and Post (24 hr) Device Attachment with varying gelation parameters (B) Contraction rate of hydrogel inserts with varying gelation conditions (C) Loss of mass of hydrogel over different time periods of attachment (D) Schematic of the silk hydrogel’s dynamic nature during device attachment. (E) Images of two HRP silk hydrogels. One of the hydrogels was cast with frog water (FW) in its gelation solution, while the other hydrogel had only distilled water (diH_2_O) in its gelation solution. Images are taken at time of gelation, and one hour after gelation. (Statistical significance: * for p < 0.05, ** for p < 0.01, and *** for p < 0.001).

The device utilizes a hydrogel insert to manipulate the wound-bed environment. This is in contrast to previous devices, which employed a solution reservoir [20]. The hydrogel insert can be removed without harm to the animal or destruction of the hydrogel. Further, the hydrogels were successfully used in post attachment analysis for mechanical testing and for mass balance assays to assess swelling and degradation (Fig 2). For the typical hydrogel concentration and time of attachment, 3.0% (w/v) and 24 hours, two statistically significant trends in the hydrogel physical properties were observed. The first was an increase of stiffness of the hydrogel insert during the time of attachment (175 N/m to 250 N/m), and the second was an average loss of hydrogel volume (62% pre/post attachment), without a corresponding loss of silk polymer mass during the time of attachment. These changes in stiffness and volume affect overall regenerative outcomes due to changes in hydration state and mechanical forces at the wound site [31], thus characterization of these two physical parameters is essential. The volume loss was similar to the contraction reported for silk HRP hydrogels in phosphate buffer [26]. Instant Ocean, used to maintain the health of the frogs, has a concentration of ions similar to phosphate buffer and since the silk HRP hydrogel is anionic, it would constrict in the presence of monovalent cations.

Past work has shown that morphological outcomes at a wound site are affected by material properties and mechanical forces present during early stages of wound healing [1]. For application in regenerative medicine, it would be advantageous to be able to manipulate these properties toward useful outcomes. Stiffness and contraction are well studied properties of hydrogels [27], thus these two parameters were manipulated for the present work. It has previously been established that the contraction and stiffness of HRP hydrogels can be tuned via the concentration of silk used in the reaction and the composition of gelation solution [26]. Therefore, hydrogel inserts were formed by varying the concentration of silk (3.5% w/v or 4.0% w/v) or additives (e.g., salt) into the gelation solution (FWG). The final stiffness of the 3.5% silk hydrogel was significant compared to the 3.0% concentration and to its pre-attached stiffness (Fig 2). The 4.0% concentration showed little or no change over time. A difference in the hydrogel’s pre-attached stiffness was identified; the 3.5% (w/v) and 4.0% (w/v) were statistically stiffer than the standard 3.0% (w/v) silk for the typical 24 hour attachment. Thus, the higher concentration hydrogel insert provided a stiffer range of dynamic contraction than the 3.0% (w/v) hydrogel inserts. The stiffness affects biological pathways in living tissue [1] and since the 3.5% (w/v) stiffness could be varied over time, this provides a useful window to explore in terms of biological impact. The less variable 4% system did not provide this window to explore.

While varying concentration affected the stiffness of the hydrogel insert, it played an even greater role in contraction of the device. At 24 hours of attachment, one the major factors that influenced stiffness of the hydrogel insert was the contraction. To tune the contraction would be useful in manipulating the mechanical forces from the sleeve. By altering the silk concentration by 3% to 4% (w/v), the change in hydrogel volume could be altered in a predictable manner over a 10% range in decreased hydrogel volume, with 3.0% (w/v) at 62%, 3.5% (w/v) at 56%, and 4.0% (w/v) at 52%. The most important observation from these trials was the precision of volume loss, and thus hydrogel contraction, achieved by a given silk concentration. The other gelation parameter that was varied the composition of the gelation solution, which involved the addition of saline (FWG). Doping gelation solutions of a hydrogel alters the material properties [32]. The effect this will have varies based on the polymer backbone, the dopant used, and the post gelation environment [32]. In this situation, the addition of salt into the silk HRP gelation solution produced changes in the physical traits of the hydrogels (optical clarity and tactile features) and an accelerated rate of contraction. For example, a decrease of diameter for the diH_2_O hydrogels by 0.2 mm versus a 1.3 mm decrease for the FWG (Fig 2E).

### Biological Outcomes

**Fig 3:**
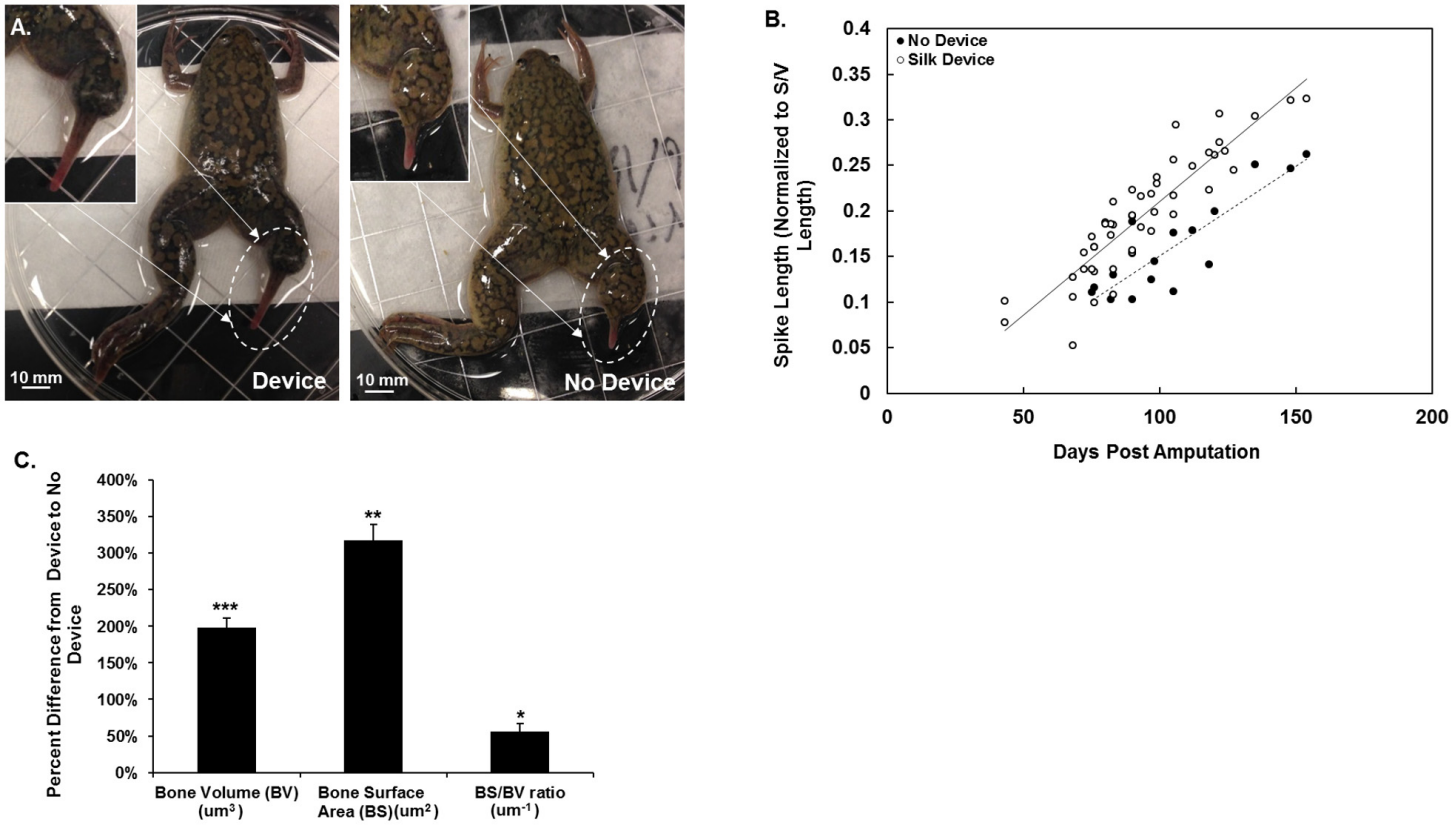
Measurements and Micro-CT Analysis of Animals. (A) Resultant spike with animals with and without device attachment (B) Non-destructive measurements of animals with and without device. (C) Micro-CT bone volume and surface area of animals that had a device attached versus those who did not. (Statistical significance: * for p < 0.05, ** for p < 0.01, and * for p < 0.001).

The physical dimensions of developing spikes in the animals were measured. A trend was observed in increased spike length in the animals with the devices (Fig 3), although this was not statistically significant. In addition, it was noted that the spikes of the animals with devices were a dark red color (Fig 3). The control animals had shorter spikes than the animals that had sleeves. Volume and surface area analysis with micro-CT was utilized to quantify the observations (Fig 3). The animals with devices had a significant increase in bone volume and surface area. The complexity of bone architecture, that is a decrease in pore size with a corresponding increase in pore number and bone density, was quantified by taking the ratio of the new bone surface area over the new bone volume. The larger this ratio, the more osteoblast/osteoclast remodeling is needed to achieve the observed porous structure in the bone [4]. There was a statistical increase in this ratio in animals with devices compared to those without (Fig 3).

**Fig 4:**
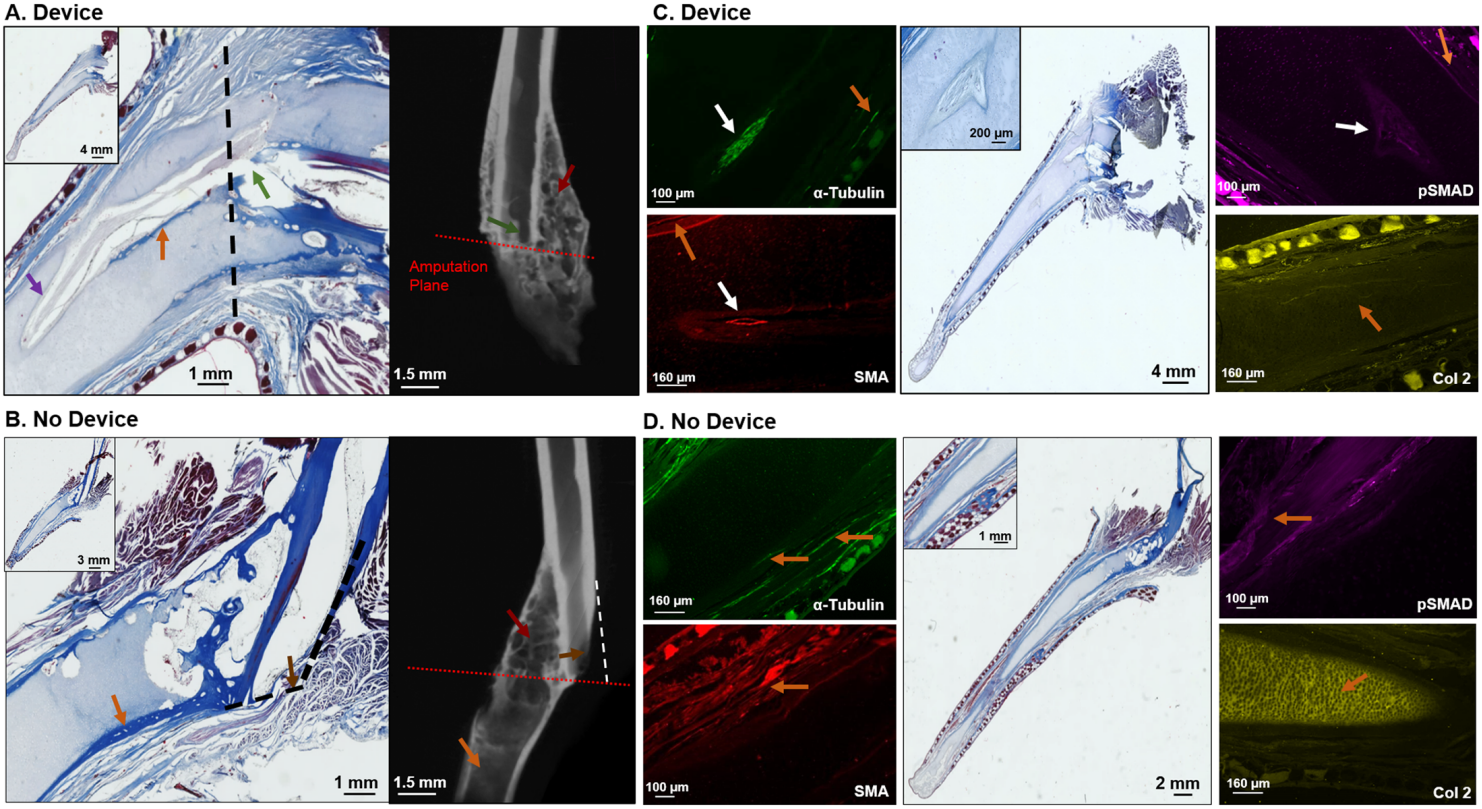
Histology, Immunohistology, and Micro-CT Images of Spike Formation from Animals. (A) and (B) Histology and Micro-CT. Red Arrows: Porous calcified tissue growth or old bone growth remodeling. Signs of osteoblast and osteoclast reconstruction can be observed in the tissues dense structure that is filled with marrow pockets. Orange Arrow: Bony callus formation, this is typically connected to the old bone around the amputation site and is hollow. Black Arrows: Formation of hyaline cartilage behind the old bone. Green Arrow: Connection site of old marrow cavity to new tissue growth. Brown Arrow: Signs that the old marrow cavity did not connect with the new tissue growth. Purple Arrow: Invasion of tissue into the cartilage spike core. (C) and (D) Immunohistological Stains. Green: Acetyl- N-Alpha Tubulin (α-Tubulin) (Nerve) Red: Smooth Muscle Actin (SMA) (Vasculature) Yellow: Collagen type II (Col2) (cartillage type II only found in abundance in hyaline cartilage) Purple: Phosphorylated Mothers Against Decapentaplegic Homolog (pSMAD 5/9) (TGF-β activator, signs of proliferation and differentiation/ not cartilage behavior) Orange Arrow: Location where protein would be typically found in spike. White Arrow: Location where protein would not be typically found in spike.

The histology and micro-CT images presented in Fig 4 compare differences between animals with and without devices. The micro-CTs for both device and control animal subgroups showed signs of calcified tissue. However, the ratio of bone surface area to bone volume was much higher in the animals with devices (device animals) compared to those without the device (controls). This outcome points to a more developed calcified tissue in the device animals. The tissue from the controls appeared to be hollow, similar morphologically to a fibrocartilage callus. The calcified tissue in the device animals was denser and featured smaller pores. While the controls also exhibited porous structures, in the device animals the porous architecture was present throughout the calcified tissue. The marrow cavity of the old bone was open to the new bone growth in the device animals but not in the controls. In the controls, the bone at the amputation site had been degraded, while in the device animals the old bone had been remodeled. The histology for the tissues from the subjects with the devices demonstrated this trend with atypical tissue formation behind the old bone. This tissue was morphologically similar to the hyaline cartilage seen in the spike core of the animal (Fig 4).

To evaluate whether the morphological changes observed in the device animals were pro-regenerative responses, immunohistochemistry was performed (Fig 4C and 4D). Markers were used for: proteins associated with innervation (N-acetyl-alpha-tubulin or α-tubulin), muscle cells used in the vasculature structure in angiogenesis (smooth muscle actin or SMA), collagen expressed at high levels in hyaline cartilage (collagen type II or col2), and a TGF-β activator associated with proliferation and differentiation (mothers against decapentaplegic or pSMAD1/5/9). The typical cartilage spike center of *adult X*. *laevis* is composed of hyaline cartilage [15]. This type of tissue is devoid of nerve and vasculature, and the cartilage cells in this tissue are not proliferative in nature. Thus, any expression of SMA, α-tubulin, or pSMAD 1/5/9 should not be seen in this tissue. Therefore, while the controls exhibited protein expression for α-tubulin, SMA, and pSMAD1/5/9 in the peripheral tissues surrounding the spike core, there was no sign of positive staining for these proteins in the cartilage center (Fig 4). The outer tissue of the spike was primarily composed of endothelial tissue, mucosal glands, and fibrocartilage, all of which would typically contain these proteins. In contrast, the cartilage center of the device animals displayed positive staining the three given antibodies.

The fourth presented antibody, collagen type II, was detected in the cartilage center of the controls, but not the device animals. Collagen type II is one the two major components found in the typical extracellular matrix (ECM) of hyaline cartilage [4]. The presented image for col2 in the control animal (Fig 4) is what is generally observed in the *normal X*. *laevis* spike formation, that is a distinct increase in fluorescence intensity for col2 in the spike center compared to the peripheral tissue [17]. On the other hand, the staining profile for the device animal shows little or no increase of intensity at the core compared the peripheral tissue. This would suggest that animals that wore the device exhibit a major decrease in one of its major ECM components compared to the controls.

### Biological Outcomes in Response to Changes in Technical Design

**Fig 5:**
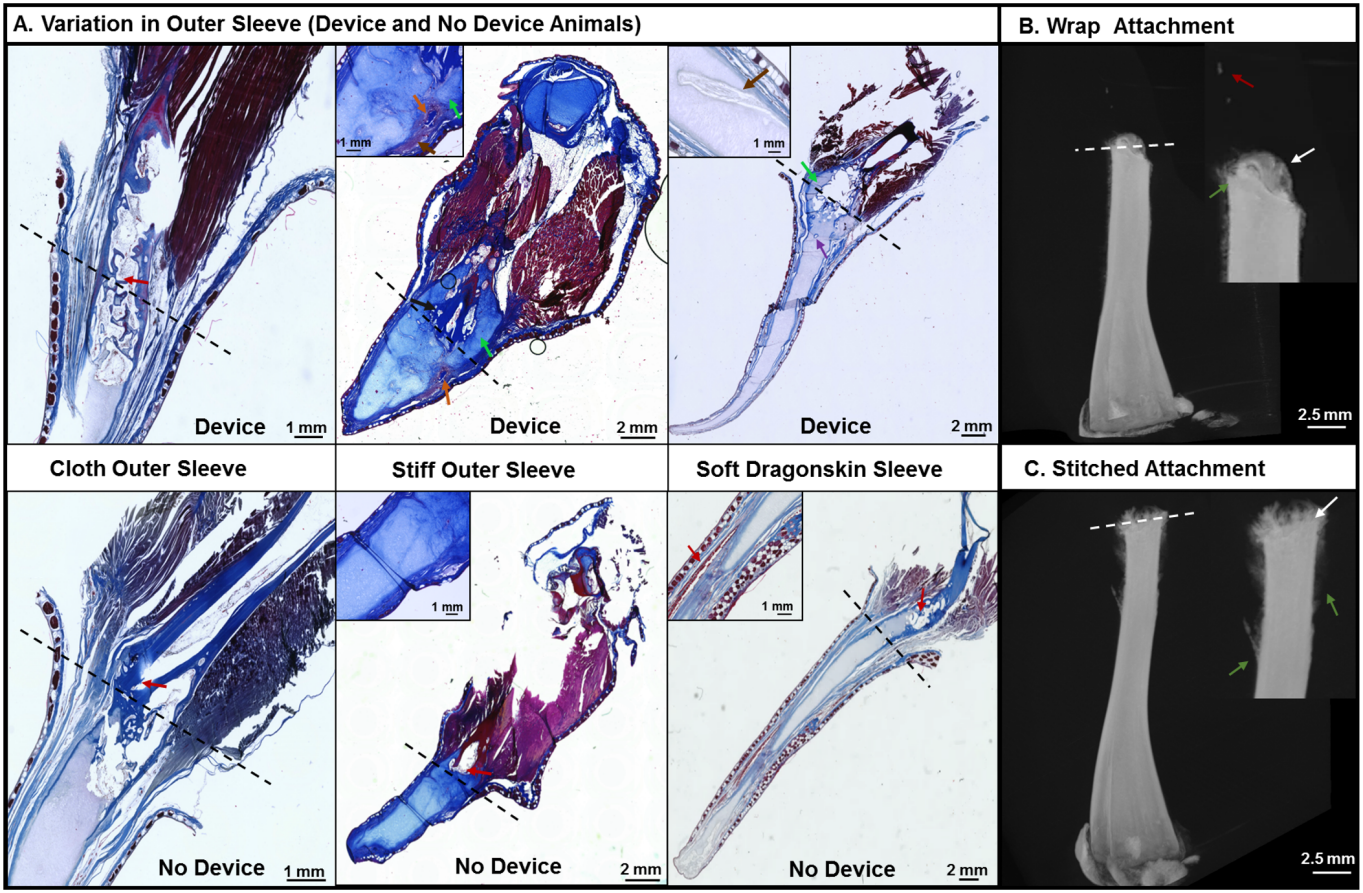
Histology and Micro-CT of Animals with Variations in Technical Design. (A) Variations Observe in Different Outer Sleeve Design. Red Arrow: Signs of Spike core isolation Brown Arrow: Connection of center cartilage tissue with without outside tissue. Orange Arrow: Ectopic Bone Growth. Purple Arrow: Bone Marrow Pocket. Green Arrow: Cartilage tissue behind the old bone. (B) Variations Observed in Different Modes of Attachment. Red Arrow: Ectopic (beyond plane of amputation) calcified tissue formation Green Arrow: Calcified tissue behind site of amputation White Arrow: Calcified Tissue at the site of amputation.

Fig 5 shows the histology of animals with the three different types of outer sleeves implemented in the device design. It should be noted that typical spike formation *for X*. *laevis* will have a hyaline cartilage center with peripheral tissues composed of skin, mucosal glands, and fibrocartilage [15]. The different types of tissue observed in typical spike formation have been well documented histologically [14,15,33], and the histology from these early studies was used as an aid in identifying the observed tissue types. The spike formation of the device animals where a cloth sleeve was used had higher calcified tissue volume and surface area than cloth sleeve control animal, but were otherwise morphologically similar to the controls. This was reflected in the decrease in new bone surface area to volume ratio for the cloth device animals in comparison to controls (Table 1). The stiff outer sleeve design showed differences morphologically to the other device designs (Fig 5). The key differences were that the animals wearing the devices showed atypical tissue formation in the tissue around the old bone growth and in the hyaline cartilage spike. These spikes were shorter and thicker at the base than in the controls. Histologically the soft dragon skin device resulted in longer and narrower spike formation as well as more tissue integration between the spike cartilage core and the surrounding tissue [15].

**Table 1:** Differences in the Calcified Tissue of the Device Animals versus their Controls.

Design Type	Calcified Tissue Volume (μm^3^)	Bone Surface Area (μm^2^)	Bone Surface Volume Ratio (μm^-1^)
Older Design (ave)	**77%**	**99%**	**15%**
*Cloth (n = 1)*	91%	77%	-7%
*Stiff Sleeve (n = 2)*	69%	111%	26%
Dragonskin Design (ave)	**255%**	**476%**	**312%**
*Thick Walls with Shorter Hydrogel inserts*	89%	110%	12%
*Thin Walls with Longer Hydrogel inserts*	380%	751%	537%

The current method of attachment used both a rubber wrap and two surgical stitches to secure the device to the amputation site with minimal damage to surrounding tissue. However, in order to understand the effect that this method of attachment on biological outcomes, both methods were evaluated individually. Thus, the devices were attached to the animals for 24 hours using either a wrap or two stitches. Micro-CT was used to image changes in calcified bone formation at 2 months post amputation. Both methods led to some calcification and showed signs of calcified tissue forward of the amputation plane. The stitching attachment method showed more calcified tissue behind the amputation plane than the wrap. The wrap attachment showed signs of calcified tissue formation within the new tissue of the cartilage spike. A small domain of calcified tissue was observed in the spike formed (Fig 5) which was confirmed using micro-CT volume analysis and is well beyond the amputation plane.

**Fig 6:**
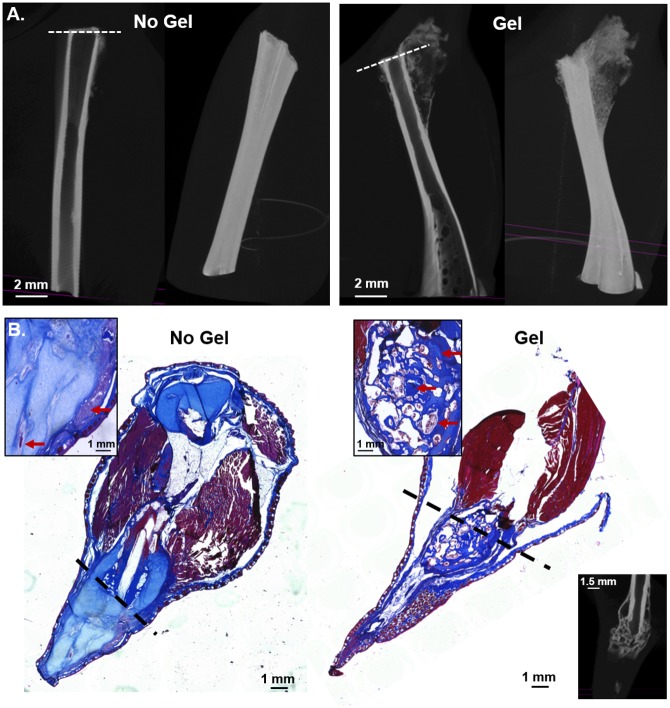
Morphological Differences in Device Animals with (Hydrogel) and without (No Hydrogel) Hydrogel inserts. (A) Micro-CT of the change in calcified tissue in two animals in the current soft Dragonskin generation of the device. (B) Comparison of resultant morphological outcomes with a stiff outer sleeve device, with and without the hydrogel insert. The hydrogel animal has an additional Micro-CT image in order to confirm that the observed changes in the histological stain are not due to tissue processing error. The dotted line represents the point of amputation.

One of the major changes in device design was the addition of the hydrogel inserts (Fig 6A). Micro-CT was used to compare calcified tissue formation between a device animal with no hydrogel insert (Fig 6A, no hydrogel) and a device animal with a hydrogel insert (Fig 6A, with hydrogel). At about 2 months post device attachment the device animal (for this situation it was a control and an experimental animal) with the attached hydrogel insert device had more calcified tissue compared to the control. Volume analysis estimated bone density of the calcified tissue growth at 0.00022 μm^-1^ and 0.00399 μm^-1^ for no hydrogel and hydrogel, respectively. The morphological difference that resulted from the addition of the hydrogel insert to the hydrogel base was also characterized (Fig 6B). The animals had the same stiff outer sleeve and utilized the wrap method of attachment. The animal in Fig 6B with hydrogel had only a hydrogel base, while the animal in Fig 6B hydrogel had both a hydrogel base and sleeve. Micro-CT was also compared with the histology. The biggest difference was at the base of the spike; in Fig 6B, no hydrogel the amputation site was clearly defined with dense pockets of calcified tissue growth, while the hydrogel animal showed a large mass of disorganized bone marrow like tissue, calcified tissue, and hyaline cartilage.

## Discussion

A device was generated that could be attached *to X*. *laevis* to study limb regeneration in a controlled setting, and without damage to the surrounding healthy tissue. Utilizing a hydrogel system as the medium of microenvironment manipulation negated the need for a water tight seal, which was the main cause of necrosis in past work [20]. The method of attachment, which utilized both stitching the device to the animal and then further securing it with a wrap, allowed for secure attachment while minimizing physical damage caused by compression.

A hydrogel insert provided important options for the device and in future work. Specifically, the hydrogel provides mechanical force, a material interface, and a substrate for drug delivery. All three of these pathways of micro-environmental manipulation can affect the final outcomes of regeneration [1,3,7]. In the present work, the hydrogel was able to handle the physical forces placed on the hydrogel during device attachment. This is due to the enzymatic crosslinks, which provided elastic behavior. This contrasts with the prior system that utilized a liquid reservoir [20]. Moreover, the different material and mechanical properties the hydrogel provides to the wound bed can be easily tuned by varying the concentration of silk or the silk molecular weight [26]. This tunability can be applied to both the static and dynamic aspects of the hydrogel insert. While the final stiffness did not change significantly by varying the silk concentration used in the hydrogel in the present work, due to the limited ranges studied, the pre-attached stiffness and degree of contraction did change and this can have a significant effect on the biological outcomes. The dynamic aspects of the different materials and mechanical properties also play an important role in the regenerative process [34]. In particular, in skin injuries the amount of pressure a bandage applies and how that pressure changes over time define initial wound closure as well as the degree of scar formation [35]. Thus, it is critical to have dynamic control over the device mechanical properties. Past work with silk HRP hydrogels has shown that changing the molecular weight can have the greatest impact on the mechanical stiffness of the hydrogel [26].

Due to the nature of the crosslinking and the natural anionic nature of the silk protein, the resultant hydrogel was sensitive to changes in pH, buffer solution, and the ionic content of the environment. This result in hydrogel insert contraction observed during device attachment. The contraction is a fundamental property of charged hydrogels and so this is not a surprising result. However, this contraction will influence the biology and should be matched to the target experimental goals. The hydrogel swelling and de-swelling can also be tuned through the molecular weight of the polymer used or a change in the composition of the solute used in the gelation process [27]. The current study explored two of the possible routes in tuning the silk HRP hydrogel insert. The overall amount of contraction was influenced by the concentration and of silk in the hydrogel as well as composition of the pre-gelation solution.

The increase in concentration of silk would have resulted in an increase in both polymer density and available reactive side chains (i.e. tyrosine) in the silk hydrogels. Both of these factors would define the ability of the hydrogel to swell and contract, since both would limit the ability of silk to accommodate water in the polymer mesh [32]. However, this observed decrease in hydrogel contraction in relation to increase in concentration is probably due to an increase of bulk polymer density rather than crosslinking density. The latter option is both sterically hindered due to the heterogeneous nature of the silk polymer chain and would also require an increase in enzyme concentration to compensate for the increased number of reactive groups. Increased polymer density versus crosslinking density would explain the impact of variation in concentration on initial gel stiffness more than its final stiffness (Fig 2A). An increase in crosslink density should result in a larger increase in final stiffness than that observed [36].

The second parameter that was varied in the work to tune the contraction of the hydrogel was the composition of the pre-gelation solution. The addition of the salt in the FW. The presence of salt can effect both the structural stability of the enzyme as well as the enzymatic reactivity. Low concentrations (the amount present in FW) can stabilize the overall tertiary structure of the protein (known as salting in) [37]. This increased structural stability would increase both the enzyme ability to crosslink and its working half-life [38,39]. Salt is also known to dramatically increase the enzymatic activity of HRP in both aqueous and non-liquid environments, such as the one presented in FW [40] or at the benchtop [41]. This could account for the increase in contraction speed that was observed in those situations.

While the magnitude of increased stiffness due to contraction was not large, it was statistically and biologically significant. The resulting change in material and mechanical forces can affect cell responses during wound healing. Different key biological pathways needed in proliferation, metalloprotease activation, and cell migration for wound closure and later stages of wound healing are activated in a mechanotransductive manner [42]. *In vivo* studies showed that application of cyclic tensile force on skin flaps in rats showed an increased rate of healing based on the speed and degree of revascularization of the injured tissue [43]. Further work demonstrated that application of a tensile force had an increased impact on angiogenesis in conjunction with the up regulation of hypoxia induced factor alpha 1 [44]. This gene is known to up regulate the production of vascular endothelial growth factor 1, and other essential proteins used in regenerative pathways [45]. Future work will focus on utilizing this device to help expand the role of mechanical forces on such areas of the wound healing process.

The spike formations observed between the animals with and without devices were morphologically evaluated. Histological and antibody staining showed qualitative differences in the anatomical structure between the experimental and control animals. This was further quantified through volume analysis of the calcified tissue. Both sets of animals showed signs of calcified tissue in the spikes. However, the quality of calcification and amount differed between the two groups. First, the controls usually displayed more of a calcified callus beyond the amputation plain that showed no signs of bone remodeling. The amount of callus formed in these controls increased over time, a trend not shown in the calcified tissue formed in the experimental animals. The calcified tissue in the device animals was more complex, with a filled in porous structure that extended beyond the amputation plane. Calcification of hyaline cartilage tissue occurs when hyaline cartilage undergoes mechanical forces and the cartilage closest to the site of mechanical forces becomes structurally reinforced by forming a calcified callus [4]. This event is known as chondrocalcinosis and is an adaptive response to the environment [4,46]. This could explain the observed fibrocartilage callus formed in the control groups. The callus would also develop over time and is similar to that seen in the controls. The observed calcified tissue for the experimental group is not an adaptive mechanism alone. The architecture was similar to that observed in trabecular bone [4]. This type of bone is formed through remodeling of calcified tissue, thus vascularization and innervation are needed. Further work is needed to understand the actual biological mechanisms, yet the complexity of the calcified tissue in the device animals appears to be a pro-regenerative response, above and beyond adaptation to the environment alone.

No matter which biological pathways are utilized to induce regeneration, innervation and vascularization will be required prerequisites in the process. Vascularization provides a route for the delivery of nutrients and oxygen, and a means to remove toxic waste from the tissue [1]. In relation to limb formation, the event which initiates the transformation of cartilage tissue to calcified bone is the vascularization of the articular cartilage tissue [4]. The immunostaining here showed that the device animals stained positive for smooth muscle actin. In this anatomic location, the only cells with high expression of this type of actin would be the endothelial cells that line blood vessels. The positive stain was found deep in the spike, even beyond the location of the calcified tissue. The nerve marker also stained positive in the same location as SMA. Tissue innervation has been demonstrated to be essential for cell migration and pattern formation for limb regeneration in axolotl [47]. Monaghan et al., demonstrated that the blastema can only be formed at an innervated amputation site [48]. The blastema provides the progenitor cells that are required for limb regeneration in this species. Therefore, it follows that for limb regeneration is dependent upon innervation.

The ECM plays major roles in tissue structure, cellular communication, and cell differentiation [12]. All of these are critical factors for successful tissue regeneration [12]. Each tissue type has a particular ECM composition designed to allow that tissue to perform its given function [12]. If the ECM composition is altered, then the function and tissue type could be altered. The results indicated a possible decrease in a major component of the typical spike ECM composition, which could point to a major alteration in the overall tissue in the device animals. Immunohistology is a qualitative assessment of the changes observed in the resultant tissue. More quantitative measures will be needed before definitive conclusions can be drawn on the observed morphological changes present in the device animal group. Yet, these findings, in the aggregate, suggest that the tissue in the resultant spike for the device animals has deviated from what is typically seen in this tissue for adult *X*. *laevis* [15]. Furthermore, this shift from the norm is one that shows an increase in innervation, vascularization, and proliferation; all requirements for regeneration [13].

One of the most defining features of the experimental animals was the level of connection that the usually isolated cartilage tissue of the spike core had with the surrounding tissue. This level of connection was seen to some degree in all the animals with devices in the stiff and soft dragonskin outer sleeve versions. Furthermore, the spike core of the experimental animals was usually connected to the old bone marrow. Device animals also had cartilage formation that surrounded the old bone growth behind the amputation plane. The expression of nerve and vasculature protein markers also tended to enter the spike core through this cartilage bridge. All the pro-regenerative changes that were observed in device animals looked similar to endochrondral ossification as seen in bone fracture healing [4]. Fracture healing occurs in three major steps: inflammation, repair, and remodeling [1,4]. The initial inflammatory response is a destructive phase in which the body destroys any foreign entities and removes damaged tissue. In the following reparative phase, hyaline cartilage forms around the fractured bone site and forms a calcified shell. The cartilage tissue then undergoes vascularization and innervation which leads to calcification and then transformation to progenitor bone cells [4]. At this point the final stage of wound healing occurs in which the newly formed bone is remodeled to its final dense and porous architecture [4]. Almost every major observed difference seen in the animals with devices can be placed into the context of these fracture healing steps, and is summarized in Table 2. Recent work from Dawson et al observed similar trends between mice fingertip amputation and fingertip bone fracture healing [49]. The key to complete regeneration in adults could be linked to manipulating the biophysical cues that controls wound resolution. Future work will explore this possibility, but what should be noted is that this device provides the required platform to explore these types of questions.

**Table 2:** Comparison of Tissues of Animals with Devices Related to Events in Wound Healing.

Correlated event in Bone Fracture Healing	Stage in Bone Fracture Healing Process	Change in Morphology in Animal with Device	Relevant Figure
*Migration of Precursor Cells from the Periosteum and Bone Marrow to the Fracture site*	Reconstructed Stage	1. Bone marrow open to new growth	Fig 4A Histology (1 and 2)
		2. Morphologically, cartilage core more connected to the surrounding tissues	
*Formation of Fracture Callus*	Reconstructed Stage	1. Development of cartilage-like tissue behind old bone growth, connects to the usually isolated cartilage spike core	Fig 5A Soft Sleeve Device (1)
		2. Calcified shell that surrounds the cartilage	Fig 4A Micro-CT (2)
*Transition of the Fracture Callus into Lamellar Bone*	Reconstructed Stage	1. Invasion of isolated cartilage spike tissue by vascularized tissue	Fig 4A and 4C (1 and 2)
		2. Formation of porous calcified tissue	
		3. Ectopic calcified tissue formation	Fig 5A Stiff Outer Sleeve (3)
*Osteoblast and Osteoclast Remodeling*	Remodeling Stage	1. Calcified tissue near the base of the spike is dense and looks similar to old bone with pockets of denser tissue, typical spike core cartilage morphologically similar to marrow	Fig 6B. Hydrogel(1)

Another key aspect to consider is that the success of bone repair relies on the ability of cells from the periosteum and bone marrow to reach the fracture site [1]. In studies of limb regeneration in *X*. *laevis*, progenitor cells from the limb bud of regenerative tadpoles were delivered to the amputation site of non-regenerative froglets through the use of a fibrin hydrogel [18]. One of the aspects of the hydrogel system that aided success was that the hydrogel helped keep the wound site open and biologically active. This aided the body in making that needed connection to the marrow cavity [18]. This could be one of the underlying mechanisms behind the device pro-regenerative abilities seen in the present work as well.

The present device went through multiple variations in design. Both the mode of attachment and the outer sleeve design had an effect on spike formation, with the hydrogel insert appearing to be the predominant factor. Since the inner hydrogel insert was in direct contact with the wound site, the local environment at the amputation site will be influenced by the different material properties of that hydrogel. Future work with the device can be broken down into two parts: characterization of the biological pathways which result from the application of the device, and technical characterization and optimization of the device design to provide the best system for future research. The fundamental mechanisms that define static and dynamic nature of the HRP silk hydrogel insert with and without the device will be needed. The presented research begins to probe and classify what drives the contraction force. The hydrogel is a weakly ionic, heterogeneously cross-linked, polymer mesh that has negative charged groups. This makes it sensitive to the ionic content of the system, as seen in the contraction during attachment. Initial variations in concentrations and gelation solution show possible methods of tuning the hydrogel to manipulate both the static and transient properties. These features can be exploited to offer defined contractive forces during healing to examine these relationships systematically. As mentioned earlier, the input of such forces is known to offer positive outcomes in skin healing [3].

Due to the nature of the present study, as the focus was on device design rather than biological pathway analysis, it is unclear whether the observed morphological phenomena were caused by activation of wound healing or regenerative biological pathways. Many of the known signs of these pathways being activated can be seen in the upregulation of certain genes within the first 2 weeks of injury[1,33,50]. This can be assessed using quantitative PCR. This information would clarify the device’s direct effect on the wound site. Clarification of old versus new tissue growth at the amputation site is also required for future device optimization. Bone could be labeled using tetracycline [51], calcein [52], and Brdu [52] to provide a more objective means of categorizing what is going on morphologically in the observed biological outcomes.

In the more technical area of device design, subsequent steps will include utilizing the hydrogel insert for drug delivery. Preliminary *in situ* experiments have demonstrated typical diffusion driven hydrogel release profiles for lithium chloride and progesterone, two compounds known to promote regeneration [53,54]. The release profiles are presented in supporting material S2 Fig. Still, more sophisticated methods will be needed to obtain more precise spatial and temporal control over any type of compound delivery. Past work on other pH sensitive hydrogels have been able to accomplish similar goals using streptavidin-biotin conjugation [55,56]. Characterization of the hydrogel dynamics will also play a critical role in this area of device design as well. The type and speed of release of any loaded compound in the hydrogel insert can be impacted by how it contracts. Past work in other pH sensitive “smart hydrogels” demonstrated a biphasic release profile by such a manipulation [57]. This level of control would be a very desirable trait in future applications, and is essential in activation of regenerative bio-pathways.

Future work will utilize the drug release capabilities of the hydrogel insert to connect observed morphological changes in the resultant spike formation to potential deviations in its underlying biological mechanism. One method which this could be done is to have the hydrogel insert deliver a given antagonists or agonists of a growth factor that is known to play a major role in an established regenerative pathway (e.g. a TNF-α inhibitor during the early stages of inflammation [5]). The changes in the outcome from the addition of the compound could be assess at both the macroscopic (e.g. variations in morphological structure and protein expression) and microscope (e.g. variations in typical DNA and RNA expression) level. Comparing the resultant deviations in both its phenotypic and genetic profile would help connect what is observed morphological to what is happening mechanistically.

## Conclusions

The purpose of the work was to develop a device that can be used to probe the biological mechanisms behind limb regeneration. The device was designed for frog amputation studies and added a layer of control and analysis through the incorporation of a silk HRP hydrogel insert in contrast to prior solution reservoirs; adding both mechanical inputs to the healing process and reduced damage to native tissue due to the attachment. The device also presented an initial set of metrics with which to evaluate the observed gross anatomical outcomes, and showed that the hydrogel insert had an effect on biological outcomes, in this case spike formation from the amputated limb. While additional work is critical to categorize possible biological mechanisms involved in these outcomes, these tools allow options to tune and evaluate this system through quantitative measures.

## Supporting Information

S1 Fig Past and Present Designs for the Device. (A) First generation hydrogel device with cloth sleeve and hydrogel base. (B) Second generation stiffer body outer sleeve (red arrow) and third generation thicker Dragonskin outer sleeve design (Blue arrow). (C) Final version of the soft Dragonskin which has a uniform sleeve thickness of 1.25 mm. The purple arrow points out a device with an outer sleeve with no hydrogel insert, while the black arrow points out an experimental device with a hydrogel insert.
(TIF)

S2 Fig Release Data for Pre-Loaded Silk HRP Hydrogels. (A) 5 mM Lithium Chloride (B) 50 and 500 μ g/mL Progesterone.
(TIF)

S1 File Additional Protocols. (DOCX)

S2 File Experimental Data. (XLSX)

S1 Table Absorbency Values for Lithium for the HRP silk Hydrogel Inserts. (A) Intensity for the control standards of lithium chloride at known values. (B) The intensity of the HRP silk hydrogels.
(TIF)
