# Supplementary figures and images for: A Tunable Silk Hydrogel Device for Studying Limb Regeneration in Adult Xenopus Laevis
Source: PLoS One. 2016 Jun 3;11(6):e0155618. doi: 10.1371/journal.pone.0155618 (PMC4892606; doi:10.1371/journal.pone.0155618)

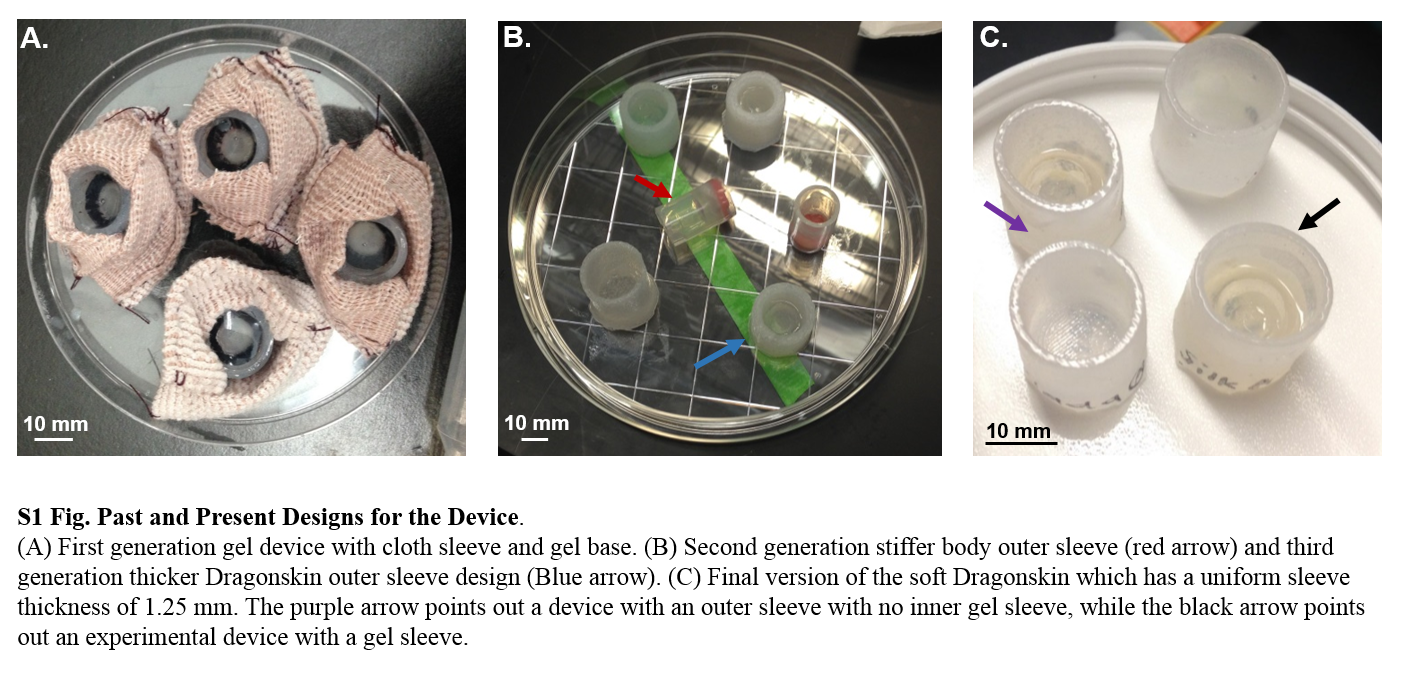

Supplement: S1 Fig — (A) First generation hydrogel device with cloth sleeve and hydrogel base. (B) Second generation stiffer body outer sleeve (red arrow) and third generation thicker Dragonskin outer sleeve design (Blue arrow). (C) Final version of the soft Dragonskin which has a uniform sleeve thickness of 1.25 mm. The purple arrow points out a device with an outer sleeve with no hydrogel insert, while the black arrow points out an experimental device with a hydrogel insert. (TIF) [file pone.0155618.s001.tif]
