# Supplementary material for: A Tunable Silk Hydrogel Device for Studying Limb Regeneration in Adult Xenopus Laevis
Source: PLoS One. 2016 Jun 3;11(6):e0155618. doi: 10.1371/journal.pone.0155618 (PMC4892606; doi:10.1371/journal.pone.0155618)
Supplement: S1 File — (DOCX) [file pone.0155618.s003.docx]

**S1 File. Additional Protocols.**

**Protocol 1. Assessment of Residual Lithium in the HRP Silk Hydrogel Sleeves using ICP-AES.**

A lithium release study was performed to assess residual lithium in the HRP silk hydrogels from the silk processing step. Eight mm diameter 3% (w/v) silk HRP gel disks were allowed to soak in 10 mL of PBS for 24 hours. For sampling, conical test tubes were inverted 8 to 10 times to ensure proper mixing, after which a 1 ml sample was taken and replaced with an equal amount of fresh dPBS. The 1 ml samples were stored at 4°C until further use. Lithium was characterized using inductively coupled plasma atomic emission spectroscopy (ICP-AES) (Prodigy Series 1000, Teledyne Leeman Labs, Hudson, New Hampshire). Samples were diluted to 2% initial concentration in nitric acid. A standard curve was calculated using a Lithium AA/ICP grade standard (Sigma-Aldrich, St. Louis) that was diluted to match a range of 10ppb to 100ppm, with an additional nitric acid solution blank. The samples were ionized under Argon gas under a magnetic field. With every trial run a set of mercury samples were also used as controls to calibrate the system. In addition, a positive control of 5 mM pre-loaded lithium chloride silk HRP gels was evaluated in parallel to the non-doped silk HRP gels and standard curve.

**Protocol 2. *In Situ* Evaluation of Progesterone and Lithium Chloride Release from the Silk HRP Hydrogel.**

Lithium chloride (Sigma-Aldrich, St. Louis, MO) release profiles were evaluated at concentrations of 5 mM. The method of release and quantification for lithium chloride followed the same procedure as described in protocol 1. Progesterone (labeled PROG in the S2 Fig.) (Sigma-Aldrich, St. Louis, MO) followed a similar release protocol as described in protocol 1, but the silk HRP gels that either contained 50 or 500 µg/mL progesterone were allowed to solidify at the bottom of the scintillation vial in which the release study took place. This allowed for a release profile more akin to the *in vivo* conditions. The progesterone release data was quantified by UV absorbance (Synergy HTX Multi-Mode Microplate Read, BioTek, Winooski, VT). The absorbance was read at 240 nm with a blank of frog water and .25 mg/mL 2-(hydroxypropyl)-β-cyclodextran (Sigma-Aldrich, St. Louis, MO) (the compound conjugated to progesterone to allow for improved solubility in water). The standard curve was set at a range of 1 to 50 µg/mL. The samples were read from a 96 well plate. Each time point had nine wells, three of which came from a separate gel’s titer for that time point. The total fraction of release was calculated based on the read absorbency and the calculated standard curve. The values were further verified with an external standard of the progesterone stock.
